# The Effect of Copper Nanoparticles and a Different Source of Dietary Fibre in the Diet on the Integrity of the Small Intestine in the Rat

**DOI:** 10.3390/nu15071588

**Published:** 2023-03-24

**Authors:** Ewelina Cholewińska, Aleksandra Marzec, Przemysław Sołek, Bartosz Fotschki, Piotr Listos, Katarzyna Ognik, Jerzy Juśkiewicz

**Affiliations:** 1 Department of Biochemistry and Toxicology, Faculty of Animal Sciences and Bioeconomy, University of Life Sciences, 20-950 Lublin, Poland; aleksandra.marzec@up.lublin.pl (A.M.); pp.solek@gmail.com (P.S.);; 2 Division of Food Science, Institute of Animal Reproduction and Food Research, Polish Academy of Sciences, 10-748 Olsztyn, Poland; 3 Department of Pathomorphology and Forensic Medicine, Faculty of Veterinary Medicine, University of Life Sciences in Lublin, 20-612 Lublin, Poland

**Keywords:** apoptosis, copper nanoparticles, dietary fibre, integrity of small intestine, inulin, pectin, psyllium, rat

## Abstract

The aim of the study was to verify the hypothesis regarding the effect of recommended (6.5 mg/kg) or enhanced (13 mg/kg) level of CuNPs in the diet in combination with different types of dietary fibre—cellulose (control), inulin, pectin or psyllium—on selected biological parameters of intestinal integrity in rats. Rats were randomly divided into 10 groups. The first two groups were fed a control diet that contained cellulose, and a mineral mixture with standard or enhanced content of CuCO_3_. Experimental groups were fed a diet supplemented with CuNPs (6.5 or 13 mg/kg) and combined with different types of fibre (cellulose, pectin, inulin or psyllium). After the feeding period, blood and small intestine samples were collected for further analysis. Replacing CuCO_3_ by CuNPs in the diet positively reduced the level of lactic acid and apoptosis markers in the small intestine; however, it also resulted in the intensification of DNA oxidation. The most beneficial effect on DNA repair mechanisms is related to inulin, while pectin has the greatest ability to inhibit inflammatory processes that induce the apoptotic death of cells in the small intestine. Our results suggest that dietary fibre supplementation protects the small intestine against potentially harmful, oxidative effects of CuNPs by intensifying the intestinal barrier.

## 1. Introduction

Copper (Cu) is a trace element involved in many physiological processes in the living organism. Importantly, Cu is a cofactor of numerous metabolic enzymes, engaged in the energy production in the respiratory chain, free radical deactivation, the maintenance of connective tissue and the proper function or modulation of nerve conduction as a result of the conversion of dopamine to norepinephrine [1,2,3,4,5,6,7]. This element also stimulates the circulatory system by iron metabolism, as well as the coagulation process and blood pressure regulation [2,6,7]. In order to ensure the proper course of the above-mentioned physiological processes in the animal’s body, it is therefore very important to provide a constant source of Cu in the diet. For this purpose, the diet is most often supplemented with a mineral mixture containing Cu at a level consistent with dietary recommendations, most often in the form of inorganic salt like CuCO_3_ [8,9,10]. However, in recent years, researchers have been giving Cu nanoparticles (CuNPs) more attention due to the specific properties determining their higher bioavailability [8,9,10]. We previously showed that CuNPs included in the diet of rats are better absorbed from the gastrointestinal tract than standard CuCO_3_ [11], but most of all, they stimulate the immune system [12] and reduce the level of DNA methylation or oxidative damage to proteins and DNA [13]. Nevertheless, others indicated that the application of CuNPs in animal nutrition, apart from the benefits, may carry a significant risk due to the potential toxicity of CuNPs [11,13,14,15,16,17]. Our results are partly consistent with these reports, as we proved an increase in Cu accumulation in the brain, kidneys and lungs, as well as an intensification of lipid peroxidation or impairment of antioxidant defence [18]. Due to such a broad physiological effect of CuNPs in the body, indicating positive and negative effects, it may be important to regulate the area of their reactivity.

Dietary fibre is one of the most important components of the diet, apart from nutrients and microelements. It is defined as parts of plants or analogous carbohydrates that are resistant to digestion and absorption in the small intestine of humans and animals, with the participation of endogenous enzymes, and are completely or partially fermented in the large intestine [19]. Dietary fibre is usually divided into soluble and insoluble fibre. Soluble fibre includes fibre which can absorb water and thus significantly increase its volume. This group includes, above all, viscous forming a gel in contact with water pectin and sticky, swelling psyllium, but also non-viscous inulin of significant prebiotic importance. The best-known example of insoluble fibre is cellulose, which does not show a significant tendency to bind water in the digestive tract [20]. Reports indicate that a diet rich in dietary fibre supports the gut microbiome and reduces the risk of cardiovascular diseases and colorectal cancer development [21]. The data also suggest that inclusion of dietary fibre in the diet may modify the processes of mineral absorption in the small intestine [22,23,24,25,26]. Moreover, the absorption of micronutrients and their impact on the intestinal tissue largely depends on the type and amount of fibre in the diet, and also minerals homeostasis in the body [27]. It has been reported that manipulation of the fibre content of the diet may have an indirect effect on Cu bioavailability by altering the bioavailability of mineral antagonists [28]. The effect of vegetable fibres on copper absorption is varied. It has been reported that dietary α-cellulose did not reduce copper absorption [29]; hence, we assumed that our choice of α-cellulose as control fibre has been justified. Different dietary fibres substantially effect the small and large intestinal luminal pH values, e.g., through the enhancement of water binding or increased viscosity, as well as the stimulation of microbiota in SCFA production. The increase in intestinal pH affects copper absorbability, presumably because of a diminished concentration of free copper. There is evidence that the fraction of Cu tightly bound to bile remains unabsorbable during its passage through the gastrointestinal tract. Some studies have shown that hemicellulose induced a negative copper balance in adolescent human males, though pectin and intact cellulose were inactive. Other refined fibres and gums, such as locust bean and karaya gums, as well as carboxymethylcellulose, have been shown to be either without effect or beneficial to the trace element balance, including copper [30].

Therefore, we assumed that the dietary combination of CuNPs with different types of fibre—neutral cellulose (control), prebiotic inulin, sticky pectin or swelling psyllium—would affect the physiological intestinal response, thereby regulating the metabolic effect of CuNPs in the body. The aim of the study was to verify the hypothesis regarding the effect of recommended Cu/kg concentration (6.5 mg) or the two times higher (13.0 mg) level of CuNPs in the diet, in combination with different types of dietary fibre (i.e., cellulose, pectin, inulin or psyllium) on selected biological parameters of intestinal integrity and histology of the small intestine in rats.

## 2. Materials and Methods

### 2.1. Chemicals and Dietary Fibre

Copper nanoparticles (99.9% purity powder, 40–60 nm size, 12 m^2^/g specific surface area (SSA), spherical morphology, 0.19 g/cm^3^ bulk density, 8.9 g/cm^3^ true density) were obtained from Sky Spring Nanomaterials Inc. (Houston, TX, USA). CuNPs with the same properties were also used in our previous studies [11,12,13,18,31]. α-Cellulose was used as a control dietary fibre source (Sigma, Poznań, Poland). The following experimental dietary fibre sources were used: pectin (PectinE 440(I), Brouwland, Beverlo, Belgium), inulin (FrutafitTex, Sensus, The Netherlands) and psyllium (Psyllim husk powder, NaturaleBio, Rome, Italy).

### 2.2. Experimental Protocol

Healthy outbred male Wistar rats 6-weeks old (Cmdb:Wi) were fed a standard semi-purified rat diet supplemented with two CuNPs doses (recommended and two times higher; 6.5 and 13 mg/kg diet, respectively) and combined with different types of dietary fibre. The control diet contained a mineral mixture with standard and enhanced content of CuCO_3_ (6.5 and 13 mg/kg diet). In the diets with Cu-NP the mineral mixture was deprived of CuCO_3_ and the copper nanoparticles were added to the diet, along with rapeseed oil (as an emulsion) for operator safety. The control dietary fibre, α-cellulose, was added at 8% of the diet, while the experimental fibre preparations (inulin with prebiotic effect, psyllium with bulk effect, pectin with viscous effect) were added at 6% of the diet at the expense of cellulose preparation (Table 1). The experimental protocol consisted of 10 groups, n = 10 per group. All animal care and experimental schema were in accordance with the Polish legislation acts concerning animal experimentation and ethical practice according to the European Convention for the Protection of Vertebrate Animals used for Experimental and other Scientific Purposes, Directive 2010/63/EU [32]. It was approved by the Local Ethics Committee for Animal Experiments in Olsztyn Local Animal Care and Use Committee (Approval No. 19/2021; Olsztyn, Poland). The study was carried out in compliance with the ARRIVE guidelines. Every effort was made to minimise the suffering of the animals used in the experiment.

### 2.3. Sample Collection and Analyses

Body weight (BW) was monitored at the start and end of the study for each animal, while the diet consumption was checked daily. Before the termination of the study, the rats were starved for 8 h but had free access to water. On the last day, the animals were subjected to body fat and lean tissue content analysis with the aid of the time-domain nuclear magnetic resonance (NMR) protocol (Minispec LF90II Bruker, Bremen, Germany). The principle of the analysis is that the tissue contrast is high between fat and muscle based on relative relaxation times. Next, the rats were anaesthetised i.p. with ketamine and xylazine in 0.9% NaCl (100 and 10 mg/kg BW, respectively) according to the anaesthesia and euthanasia guidelines for laboratory rodents. Following laparotomy, blood samples were drawn from the caudal vena cava into heparinised tubes, and finally the rats were euthanised by cervical dislocation. After that, the small intestine, heart, kidneys and spleen were dissected and weighed. Samples of the small intestine were preserved for histopathological examination, and intestinal tissue homogenates were prepared and stored at −80 °C until analysis. Blood plasma was prepared by solidification and low-speed centrifugation (350× *g*, 10 min, 4 °C). Plasma samples were kept frozen at −80 °C until assay.

The pH values in the ileal digesta were measured with the aid of a pH/ion metre (model 301, Hanna Instruments, Vila do Conde, Portugal), while the dry matter (DM) concentration in the digesta was analysed by sample drying at 105 °C to a constant weight. The apparent digesta viscosity was measured in the supernatant fraction using the cone/plate viscometer (model LVDV II+, Brookfield Engineering Laboratories, Stoughton, MA, USA).

In the blood plasma, the level of selected indicators of intestinal integrity—lactic acid (LA) and diamine oxidase (DAO)—was determined using a commercial measurement enzyme-linked immunosorbent assay (ELISA) kit, following the protocol provided by the manufacturer (Shanghai Qayee Biotechnology Co. Ltd., Shanghai, China). Absorbances were measured at 450 nm via ELISA reader (Sunrise^TM^, Tecan Group Ltd., Männedorf, Switzerland).

In homogenates prepared from the small intestine, the level of 8-hydroxydeoxyguanosine (8-OHdG), apurinic/apyrimidinic endonuclease 1 (APE-1), 8-oxoguanine DNA glycosylase (OGG1), lactic acid (LA), diamine oxidase (DAO) and the level of caspase 3 and caspase 8 were determined. These parameters were determined using a commercial measurement enzyme-linked immunosorbent assay (ELISA) kit, following the protocol provided by the manufacturer (Shanghai Qayee Biotechnology Co., Ltd., Shanghai, China). Absorbances were measured at 450 nm via ELISA reader (Sunrise^TM^, Tecan Group Ltd., Männedorf, Switzerland).

### 2.4. RNA Extraction and Quantitative Real-Time PCR

RNA from the small intestine was extracted using Trizol Reagent (Thermo Fisher Scientific, Waltham, MA, USA), according to the manufacturer’s protocol. The isolated RNA yield was estimated spectrophotometrically (UV-VIS spectrophotometer Nabi, MicroDigital Co. Ltd., Gyeonggi, Republic of Korea), with integrity assessed electrophoretically by separation on 0.8% agarose gel. For complementary cDNA synthesis, 1 µg of RNA was reverse transcribed using the NG dART RT kit (EURX Ltd., Gdańsk, Poland), according to the manufacturer’s instructions. Specific primers for evaluation of genes expression—zonula occludens-1 (*ZO-1*), occludin (*OCLN*), trefoil factor 2 (*TFF2*) and 8-oxoguanine glycosylase (*OGG1*)—were designed using Primer 3 software (Whitehead Institute, Cambridge, MA, USA) and synthesised by Genomed (Warsaw, Poland). The sequences are shown in Table 2.

Real-time PCR was performed on Quantabio thermocycler (VWR International LLC, Radnor, PA, USA) and using SG qPCR Master Mix (2×) (EURX Ltd., Gdańsk, Poland) according to the following protocol: one cycle at 95 °C for 10 min (initial denaturation), followed by a PCR including 35 cycles at 95 °C for 20 s (denaturation), 59–62 °C for 20 s (annealing) and 72 °C for 30 s (elongation). A melting curve analysis was performed over 50–72 °C at 0.3 °C/s intervals. Negative controls without the cDNA template were provided. Real-time PCR was performed in duplicate. Normalised gene expression was calculated using the 2^−ΔCt^ method. The glyceraldehyde-3-phosphate dehydrogenase (*GAPDH*) and β-actin (*ACTB*) genes were selected as endogenous controls to normalise gene expression.

### 2.5. Histological Examination of Organs

Histopathological examinations of small intestine samples from rats were performed according to the procedure described by Cholewińska et al. [31].

### 2.6. Data Analysis and Statistics

The STATISTICA software, version 12.0 (StatSoft Corp., Krakow, Poland), was used to determine the differences among treatment groups. Two-way ANOVA was applied to assess the effects of main factors CuNPs dose (L, 6.5 mg/kg and H, 13 mg/kg) and dietary fibre type (cellulose, pectin, inulin and psyllium), followed by Duncan’s multiple range test. Additionally, each experimental group fed CuNPs L dose was compared with the control C group (fed diet with 6.5 mg/kg Cu from CuCO_3_ and containing cellulose as the main dietary fibre source) with the aid of a *t*-test. Similarly, the *t*-test was used to compare the experimental groups fed diets with CuNPs H dose with the control CH group fed diet with 13 mg/kg Cu from CuCO_3_ and containing cellulose as the main dietary fibre source. Differences with *p* ≤ 0.05 are considered to be significant.

## 3. Results

### 3.1. One-Way Analysis of Variance (ANOVA)

#### 3.1.1. C vs. CN, PN, JN and SN

(The C group was fed a control diet with standard Cu content in the mineral mixture (6.5 mg/kg) from CuCO3 with 8% of cellulose as dietary fibre source; the CN group was fed a diet with a supplementation of 6.5 mg Cu/kg from CuNPs with 8% of cellulose dietary fibre source; the PN group was fed a diet with a supplementation of 6.5 mg Cu/kg from CuNPs with 2% of cellulose and 6% of pectin dietary fibre source; the JN group was fed a diet with a supplementation of 6.5 mg Cu/kg from CuNPs with 2% of cellulose and 6% of inulin dietary fibre source; and the SN group was fed a diet with a supplementation of 6.5 mg Cu/kg from CuNPs with 2% of cellulose and 6% of psyllium dietary fibre source).

At the beginning of the experiment, a significant reduction in daily weight gain was observed in the JN and SN groups. The JN group also showed a significant reduction in daily dietary intake, with a simultaneous increase in kidney weight compared to the control (Table 3). There was also an increase in the weight of the small intestine with its content in the PN, JN and SN groups; the ileal viscosity in the PN and SN groups; as well as the tissue and contents weights of the cecum in the JN and SN groups. Reduced ileal DM was also observed in the PN and JN groups compared to the control. In the PN group, a lower pH of the ileum content was found, while in the SN group, the value of this parameter was increased compared to control (Table 4). An increased DAO activity in serum was also found in the JN group. In the blood plasma of all compared experimental groups (CN, PN, JN and SN), a reduced content of lactic acid was noted (Table 5). In the tissue of the small intestine of PN group rats, a significant decrease in the level of APE-1 was noted, while in the CN group, the level of lactic acid was significantly reduced compared to the control. In the small intestine tissue of rats from all compared experimental groups (CN, PN, JN and SN), reduced levels of Caspase-3 and Caspase-8 were demonstrated (Table 6). Moreover, a decrease in the level of expression of the *OGG1* gene in the CN, JN and SN experimental groups; the *TFF2* gene in the CN, PN and SN experimental groups; and the *ZO-1* gene in the CN, PN and JN experimental groups compared to control group was found (Table 7).

#### 3.1.2. CH vs. CNH, PNH, JNH and SNH

(The CH group was fed a control diet with enhanced Cu content in the mineral mixture (13 mg/kg from CuCO_3_) with 8% of cellulose as a dietary fibre source; the CNH group was fed a diet with a supplementation of 13 mg Cu/kg from CuNPs, with 8% of cellulose as a dietary fibre source; the PNH group was fed a diet with a supplementation of 13 mg Cu/kg from CuNPs with 2% of cellulose and 6% of pectin as a dietary fibre source; the JNH group was fed a diet with a supplementation of 13 mg Cu/kg from CuNPs with 2% of cellulose and 6% of inulin as a dietary fibre source; the SNH group was fed a diet with a supplementation of 13 mg Cu/kg from CuNPs with 2% of cellulose and 6% of psyllium as a dietary fibre source).

Next, a significant increase in spleen weight was observed in the CNH and JNH groups (Table 3). In addition, a significant increase in the weight of the small intestine content and a decrease in the ileal DM in the PNH, JNH and SNH groups was noted. Compared to the CH group, the ileal viscosity in the PNH and SNH groups was increased. Moreover, an increase in the pH of the ileum content was noted in the SNH group (Table 4). Decreased lactic acid level in the serum was noted in the PNH and JNH groups vs. CH (Table 5). In all experimental groups (PNH, JNH and SNH), an increase in the level of OGG-1 in the tissue of the small intestine was observed. Moreover, in the examined tissue of rats from the JNH group, a decrease in the level of APE-1 was also noted, with a simultaneous decrease in the content of lactic acid compared to the CH control group. A reduction in the content of Caspase-8 was also found in the small intestine of rats from the CNH group vs. the CH group (Table 6). In the small intestine of SNH rats, an increase in the expression of *OCLN* and *TFF2* genes was observed, while a decrease in the expression of the *OGG1* gene was noted compared to CH control group. Intestinal *TFF2* gene level in the PNH group and *ZO-1* gene expression level in the PNH, JNH and SNH groups were higher compared to CH control (Table 7).

### 3.2. Two-Way Analysis of Variance (ANOVA)

The interactions for plasma lactic acid content were noted (*p* < 0.001; Table 5), as well as APE-1 and lactic acid levels (*p* = 0.020 and *p* = 0.019, respectively; Table 6) and *OGG1* gene expression (*p* = 0.037; Table 7). The occurrence of these interactions indicates that the main effects did not have a significant effect on the parameters that were studied. The interaction observed for the level of lactic acid (*p* < 0.001) in the blood plasma of rats is related to the fact that the use of a higher dose of CuNPs resulted in an increase in the content of this indicator in rats from the CN group, which was not observed when using a higher dose of CuNPs in the PN, JN and SN groups (Table 5). The interaction observed for the level of APE-1 (*p* = 0.020) in the tissue of the small intestine of rats is due to the fact that the use of a higher dose of CuNPs resulted in an increase in the level of this enzyme in rats from the PN, JN and SN groups, which was not observed with the use of higher dose CuNPs in the CN group. In turn, the interaction observed for the content of lactic acid (*p* = 0.019) in the small intestine of rats is related to the fact that the use of a higher dose of CuNPs resulted in a decrease in the content of this indicator in the PN and SN groups, which was not observed when using a higher dose of CuNPs in the CN and JN groups (Table 6). The interaction observed for the intestinal *OGG1* expression level (*p* = 0.037) is related to the fact that the use of a higher dose of CuNPs resulted in a decrease in these parameters in rats from the PN group, which was not observed when using a higher dose of CuNPs in the CN, JN and SN groups (Table 7).

#### 3.2.1. Effect of CuNPs Dose

The introduction of twice as high levels of CuNPs into the diet did not have a significant effect on the increased level of Cu in the blood plasma, because the values recorded in individual groups were in the range of 89.9–111 µmol/l (unpublished data). Increasing the level of CuNPs from 6.5 to 13 mg/kg diet in the rat diet resulted in an increase DAO levels in the blood (*p* = 0.001; Table 5). 

#### 3.2.2. Effect of Dietary Fibre Type

Further, it was observed that feeding rats with a diet containing inulin or psyllium resulted in a decrease in daily weight gain and daily dietary intake (*p* = 0.028 and *p* = 0.004, respectively) compared to the control receiving cellulose as a standard dietary fibre source (Table 3). Importantly, the results were independent of the level of CuNPs used. Including pectin, inulin or psyllium fibres resulted in an increase in the weight of the small intestine and its contents, while reducing the ileal DM (*p* < 0.001, both) compared to cellulose only. The addition of pectin or inulin lowered the pH of the ileal contents of rats (*p* < 0.001) also. Supplementation of pectin or psyllium also resulted in an increase in ileal viscosity (*p* < 0.001; Table 4). In addition, reduced levels of DAO were found in the blood serum of rats fed a diet supplemented with inulin or psyllium (*p* = 0.036; Table 5). Including inulin resulted in an increase in the level of OGG-1 (*p* = 0.035), while the inclusion of pectin to the diet contributed to a decrease in the level of caspase-3 (*p* = 0.043) in the small intestine compared to being control fed a diet containing cellulose only (Table 6). In the small intestine of rats receiving a diet enriched with psyllium, a higher level of *ZO-1* (*p* = 0.013) gene expression than in the control rats was also observed (Table 7).

### 3.3. Histology Examination of Small Intestine

**Figure 1:**
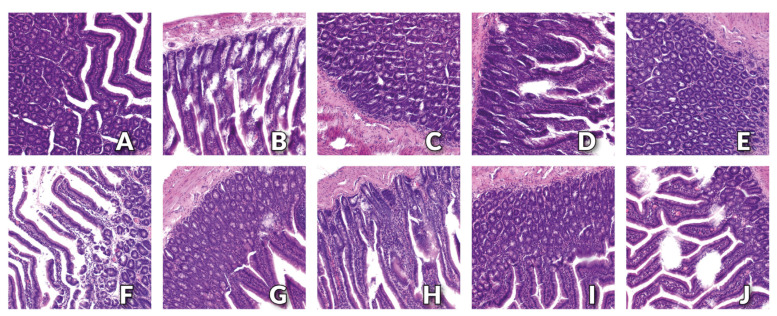
Morphological effects of different Cu sources (CuCO_3_ or CuNPs), CuNPs levels (L—6.5 mg Cu/kg diet or H—13.0 mg Cu/kg diet) or type of fibre (**C**—cellulose, P—pectin, J—inulin or S—psyllium) in the diet on the small intestine of rats; 10× magnification. Treatments: (**A**)—C, (**B**)—CH, (**C**)—CN, (**D**)—CNH, (**E**)—PN, (**F**)—PNH, (**G**)—JN, (**H**)—JNH, (**I**)—SN, (**J**)—SN.

The histopathological examination showed a physiological structure of the small intestine with the presence of small–insignificant tissue defects in the apical parts of the villi in rats from C and CH groups (Figure 1A,B, respectively). The normal, physiological structure of the small intestine was found for the CN and PN groups (Figure 1C,E, respectively). In the small intestine of rats from the JN and SN groups, single–significant tissue defects in the apical parts of the villi were found (Figure 1G,I, respectively). In the small intestine of rats from the CNH, PNH, JNH and SNH groups, damage to the apical part of the villi was numerous and significant, wherein these lesions were the most severe in the CNH group (Figure 1D,F,H,J, respectively). No pathological changes at the base of the villi and in the mucosa of the small intestine were found in any of the examined groups of rats.

## 4. Discussion

The results of the study did not show that the replacement of the recommended level of Cu by CuNPs affected the diet intake and body weight of experimental rats, which is fully consistent with the results of our previous studies on rats [11]. There were also no significant changes in the analysed growth parameters in response to the increasing CuNPs level. However, a decrease consumption and body weight were noted in rats whose diet was supplemented with inulin or psyllium. Others also report such observations [25,34,35,36,37,38,39]. An explanation may be that dietary fibre naturally increases the feeling of satiety, consequently reducing the overall caloric intake [37]. The observed effects caused by inulin or psyllium may be related to the fact that both forms dissolve well in water, forming a gel in the small intestine, which prolongs the feeling of satiety, thus reducing the intake of energy and weight loss, or to the specific rheological properties of high-fibre foods that require longer chewing [40]. Adam et al. [37] prove that soluble dietary fibre of a prebiotic nature stimulates the secretion of satiety hormones, such as glucagon-like peptide-1 (GLP-1) and tyrosine-tyrosine peptide (PYY), by the mucosa of the small intestine, which effectively suppresses appetite and reduces the food intake. Moreover, in their studies, the authors have also shown that the stimulation of the secretion of satiety hormones may occur as a result of the activation of receptors located in the small intestine by an increased amount of signalling short-chain fatty acids (SCFAs) produced during fibre fermentation [37]. Interestingly, despite the significant role of pectin in dietary intake and body weight reduction [37], the results of our study did not confirm the significant effect of this form of fibre on the reduction of growth parameters in rats. In turn, our results are consistent with others [41,42], and proved that a diet containing pectin is well tolerated, and no changes in the amount of food consumed or body weight were noted. Taken together, the results presented here and elsewhere suggest that the observed effects are correlated with dietary pectin levels. It seems possible that the inclusion of a higher proportion of this type of fibre in the diet could result in a decrease in the growth rate of rats tested.

Further, the replacement of CuCO_3_ by CuNPs, as well as a two-fold increase in their level compared to dietary recommendations had no effect on intestinal parameters, such as small intestine weight with contents, and the dry matter, viscosity or pH of the ileum. However, these parameters can be modulated by introducing alternative forms of dietary fibre to the diet. The results of our study showed an increase in the weight of the small intestine with its content in rats fed a diet containing pectin, inulin or psyllium. Our results are consistent with others [42,43,44,45]. Dongowski et al. [42] noted an increase in the weight of the ileum tissue and its content in rats fed a diet containing pectin. A similar increase in the weight of the small intestine in rats fed a diet containing pectin for 20 days was also noted by Pirman et al. [46]. Moreover, Krupa-Kozak et al. [45] proved that a gluten-free diet supplemented with prebiotic inulin for 6 weeks also showed a significant increase the relative weight of the small intestine in rats. Arjmandi et al. [43] observed an increase in the relative weight of the small intestine in rats fed a diet containing cholesterol and 5 or 10% psyllium for 21 days, which was stored at 5 °C or 40 °C for 8 months, compared to a control group receiving a diet containing 10% cellulose. Kristensen et al. [44] also observed analogous changes as a significant increase in the weight of the gastrointestinal tract in total and its individual segments, including the small intestine, in rats receiving a dietary fibre supplement from linseed. Dongowski et al. [42] explain the obtained effect by the fact that dietary fibre (especially pectin) is not or only slightly degraded in the small intestine of rats, which means that the fibre contained in the gastrointestinal content binds water, thereby increasing its viscosity. The small intestine has a difficult task because it has to transport this sticky content to the lower segments of the digestive tract, which causes its cells to grow, and, as a consequence, its mass also increases. Although our study confirmed an increase in ileal viscosity in rats fed a diet supplemented with pectin and psyllium, histological evaluation did not reveal an overgrowth of the small intestinal wall cells in any of the experimental groups. In our study, an increase in the acidity of the ileum of rats fed a diet with the supplementation of pectin and inulin was also shown. Krupa-Kozak et al. [45] suggest that an increase in the weight of the small intestine with simultaneous acidification of its environment may indicate that inulin (and perhaps other soluble forms of fibre) may be metabolised to some extent by air-tolerant small intestine bacteria. The increased weight of the small intestine with its content obtained in our research, with a simultaneous reduction in the dry matter of the ileum in rats from all experimental groups, and an increase in the viscosity of the small intestine of rats receiving pectin or psyllium, allows us to assume that the observed changes may be primarily the result of hydration properties of dietary fibre. All three forms of tested dietary fibre (inulin, pectin and psyllium) have a tendency to dissolve in water and bind it, wherein pectin forms very viscous solutions, and psyllium swells when binding water [37]. Food containing fibre, passing through subsequent sections of the digestive tract, up to the small intestine, binds water while increasing its volume and weight. Taking into account the significant increase in the total weight of the small intestine with the simultaneous decrease in the dry matter of the ileum in all experimental groups, it may be assumed that these differences were affected by the weight of water bound by dietary fibre present in the intestinal content.

In the assessment of the intestinal barrier function, diamine oxidase (DAO) is often considered. This enzyme catalyses the decomposition reaction of histamine in the digestive tract, which is responsible for the development of an allergic reaction. Under physiological conditions, a relatively high level of DAO is found in the small intestine, while in blood plasma it is very low. When the intestinal mucosa is damaged, e.g., as a result of ischemia, hypoxia, contact with a harmful factor present in food or tissue nutrition disorders, an inflammatory reaction is triggered with the release of histamine. Due to the necessity of its deactivation, a decrease in the level of DAO in the intestinal mucosa is observed, resulting in a simultaneous decrease in the level of this indicator in the blood [47]. Our results indicate that the replacement of Cu in the standard form of CuCO_3_ by an equivalent dose of CuNPs had no effect on the DAO level in the blood plasma and small intestine. However, the level of DAO in the blood plasma increased with no changes in the level of this indicator in the small intestine as a result of increasing the CuNPs dietary level. Surprisingly, the results of our study also showed no significant differences in the level of intestinal DAO between the experimental groups and control group fed a diet containing the addition of cellulose, while the value of this indicator was significantly increased in the blood plasma of rats receiving a diet containing the addition of inulin or psyllium. Although the obtained results are ambiguous, they undoubtedly indicate a beneficial effect of both the introduction of a doubled dose of CuNPs compared to the standard recommendation, as well as alternative forms of fibre (inulin and psyllium) on maintaining a proper intestinal barrier.

Lactic acid is the end product of glucose oxidation in anaerobic glycolysis [48]. It may be synthesised in situ on the intestinal mucosa by anaerobic bacteria colonising the lumen of the large intestine, such as lactobacilli, streptococci and bifidobacteria [49,50]. There are reports that this compound may affect a number of metabolic and immunological processes in the body, including mediation in the signalling pathways; the production of pro- and anti-inflammatory mediators by T lymphocytes and macrophages; or by affecting the redox status through the reaction of lactate dehydrogenase inducing reactive oxygen species and acting as an inhibitor of glucose breakdown [49]. Okada et al. [51], in studies on mice, also proved that lactate can stimulate the proliferation of enterocytes, which has a positive effect on maintaining the function of the intestinal barrier. However, there are also studies that found an increase in the synthesis of lactic acid in the intestines and the accompanying discharge of this compound into the blood, which may be observed in the case of intestinal hypoxia, resulting in the intensification of anaerobic glucose breakdown. As a consequence, it may lead to dangerous lactic acidosis development [48]. The results of our study indicate that replacing the traditional CuCO_3_ with CuNPs resulted in a reduction in the level of lactic acid in the small intestine and blood serum as well. In light of the above-mentioned reports, this effect may be considered positive. In addition, the increased lactic acid level in the intestine may result in acidification of the environment. In the case of the large intestine, this effect may be desirable, because it protects it, among others, from colonisation by pathogenic bacteria [52], while, in the case of the small intestine, this effect may be completely different. Digestive enzymes secreted into the lumen of the small intestine require a slightly alkaline environment for their activation and proper functioning [53]. Acidification of the environment caused by the overproduction of lactic acid in the small intestine may therefore result in a reduction in digestive processes, and thus, in the efficiency of the absorption of nutrients. This also seems to confirm the beneficial effect of CuNPs on the functioning of the small intestine. Moreover, our study also noted a significant DxF interaction for LA. This results from the fact that, in the case of the combined use of pectin or psyllium as a source of fibre with a higher level of CuNPs, a reduced level of this indicator was found in the intestinal wall, which was not observed in the case of the combined use of nanoparticles with cellulose or pectin. It is likely that the use of a higher level of CuNPs with the above-mentioned sources of fibre has a more beneficial effect on intestinal integrity than its combination with cellulose, as also indicated by the increased level of LA in the blood plasma of rats from the cellulose group, together with a higher level of CuNPs in the diet. Increased production of lactic acid in the small intestine is an unfavourable phenomenon, as it can lead to acidosis, which results in serious damage to the intestinal epithelium, resulting in excessive intestinal permeability, referred to as “leaky gut” [54].

Among all organs, the tissues of the gastrointestinal tract, especially the small intestine, is most exposed to the potentially harmful effects of xenobiotics entering the body through diet. It is assumed that this harmful effect is very often associated with the increased synthesis of free oxygen radicals. These, in turn, contribute to the occurrence of oxidative stress, resulting in damage to cellular macromolecules, including DNA modifications such as the oxidation of nitrogenous bases [55]. In order to prevent the loss of genome integrity, the body has developed various repair systems, among which a very important role is played the DNA repair pathway by cutting out damaged bases (BER) and replacing them with correct ones. This process is initiated by DNA glycosylases, which include, among others, 8-oxoguanine glycosylase (OGG1). This enzyme removes the most common DNA damages such as 8-hydroxydeoxyguanosine (8-OHdG) and 2,6-diamino-4-hydroxy-5-formamidopyrimidine (FapyGua) [56]. The results of our previous studies showed no deterioration of the oxidoreductive status of the small intestine in rats due to the replacement of the standard form of Cu by CuNPs [31]. The highly reactive CuNPs may undergo Fenton and/or Haber–Weiss reactions in the body, resulting in the enhanced production of free radicals, which then damage the genetic material [31]. The results of the present study partially confirm that the replacement of CuCO_3_ by CuNPs resulted in an increase in the level of 8-OHdG in the small intestine, which suggests the intensification of oxidative processes in the examined tissue. Interestingly, the inclusion of the recommended level of CuNPs in the diet of rats contributed to a downregulation of *OGG1* gene expression in the small intestine, which, however, did not translate into a decrease in the amount of functional OGG1 protein. It was also not found that increasing the level of CuNPs in the diet of rats from 6.5 to 13 mg Cu/kg of diet resulted in a deterioration of the oxidoreductive status and weakening of DNA repair mechanisms in the small intestine. However, an increase in the OGG1 protein level was noted, with no changes in *OGG1* gene expression and 8-OHdG content in rats supplemented with inulin. This suggests that excessive oxidation of nitrogenous bases does not occur, and thus, the OGG1 enzyme is not significantly used for their repair. This allows us to assume that among all the tested forms of fibre, inulin best protects DNA against the harmful effects of free radicals, which may potentially be formed as a result of including CuNPs in the diet. The beneficial effect of the combined use of pectin with a higher level of CuNPs is evidenced by the observed DxF interaction for the level of APE-1 and for the expression of the *OGG1* gene in the small intestine, resulting from the fact that only in the case of combining pectin as a source of fibre with the addition of a higher level of CuNPs, an increase in the level of APE-1 and downregulation of *OGG1* were noted. Although an increase in the APE-1 level (apurinic/apyrimidinic endonuclease 1, one of the DNA repair enzymes) may indicate an increased oxidation of this acid [57]. However, due to the fact that an increased level of 8-OHdG was not observed when a higher level of CuNPs was used together with pectin compared to the use of a lower level of CuNPs with pectin, it should be concluded that the increase in the level of APE-1 was not associated with the induction of the repair mechanism.

In the event of damage to important cellular macromolecules, the programmed cell death may be activated. Caspases play an important role in this multi-stage process [58]. The results of our study indicate that the replacement of CuCO_3_ by CuNPs resulted in a decrease in caspase 3 and caspase 8 levels in the small intestine of rats, which seems to be a very beneficial phenomenon, and proves that this supplement does not induce significant negative changes in the cells of the small intestine. It also allows us to assume that the previously mentioned induction of oxidative DNA damage as a result of including CuNPs in the diet of rats was not serious enough to lead to significant changes in the genome of intestinal cells, which would result in their being directed to the apoptotic pathway. Moreover, it was not found that increasing the level of CuNPs in the diet contributed to the occurrence of negative changes in the level of the tested caspases. Regardless of the level of CuNPs introduced into the rats’ diet, no increase in the synthesis of caspases was found as a result of inulin and psyllium inclusion in the diet, and the addition of pectin reduced the level of caspase 3 in the wall of the small intestine. This suggests that, among the studied forms of fibre, pectin best protects the cells of the small intestine against damage that could lead to their apoptosis.

The intestinal barrier is crucial for maintaining intestinal homeostasis, which determines the proper supply of the body with nutrients and the prevention of intestinal diseases. It consists of the apical cell membrane and intercellular tight junctions (TJ) of enterocytes. Tight junctions are protein complexes resulting from the interaction between members of the claudin family, zonula occludens and MARVEL tight junction proteins (TAMP) [59]. ZO-1 is a cytoplasmic plaque protein that recruits various signalling molecules and acts as a scaffold for transmembrane TJ proteins [60]. This protein is encoded by the *ZO-1* gene, which is expressed especially in the intestine, kidneys, liver, lungs and brain [61]. There are reports that a decrease in ZO-1 protein expression may increase intestinal permeability, which, in turn, may result in the development of intestinal inflammation and even the development of neoplastic lesions [59,62]. It is assumed that Trefoil family factor 2 (TFF2), encoded by the gene of the same name, also plays an important role in maintaining a proper intestinal barrier [63]. The results of our study showed that *TFF2* may be expressed in the wall of the small intestine. Interestingly, the *TFF2* protein is involved in intestinal defence and repair mechanisms, while *TFF2* overexpression is often observed in the case of significant tissue damage, infection or neoplastic changes in the within the digestive tract [63]. The results of our studies indicate that the replacement of CuCO_3_ by CuNPs in the diet of rats resulted in *TFF2* and *ZO-1* downregulation, with no changes in the expression level of *OCLN*. However, there was no effect of doubling the level of CuNPs in the diet—in relation to the nutritional recommendations—on the level of expression of the analysed genes of the intestinal barrier in rats. The obtained results can be interpreted in two ways, because the downregulation of *TFF2* gene expression by CuNPs seems to be beneficial, but a decrease in the level of *ZO-1* may indicate that CuNPs increase intestinal permeability and, thus, deteriorate the intestinal barrier. The explanation of the obtained results may be related to the formation of a functional protein, which reduces the expression of the *TFF2* and *ZO-1* mRNA pools. The results of our study also showed that the inclusion of dietary fibre in the form of psyllium in the diet of rats increased the *ZO-1* gene expression in the small intestine, which, in light of reports by other authors, is an effect conducive to maintaining the proper integrity of the intestinal barrier [59,62].

Finally, the results of histopathological examination indicate the normal structure of the small intestine with the presence of small tissue defects in the apical parts of the villi in rats receiving a standard diet containing the recommended level of Cu in the form of CuCO_3_ and cellulose as a source of fibre. We observed no adverse changes in the morphology of the small intestine in rats receiving a diet containing the 6.5 mg/kg of CuNPs and the addition of cellulose (control group) or pectin. For rats fed a diet containing the same level of CuNPs but supplemented with inulin or psyllium, single tissue defects were observed at the top of the intestinal villi. Similar changes were intensified by the inclusion of a higher level of CuNPs (13 mg/kg) in the diet, regardless of the form of fibre used, with the most significant and numerous losses of the top part of the intestinal villi observed in the group receiving cellulose in the diet. The obtained results indicate that the replacement of CuCO_3_ by CuNPs had a beneficial effect on the morphology of the intestines. In turn, changes in the top parts of the villi observed in groups of rats fed a diet containing an increased level of CuNPs, regardless of the fibre source used, seem to be related to the direct effect of Cu in the form of nanoparticles. However, the lack of any pathological changes at the base of the villi and in the mucosa of the small intestine, regardless of the type of dietary fibre, as well as the lack of deterioration of indicators proving the integrity of the intestinal barrier, do not seem to confirm this assumption. Moreover, our previous studies did not show any pathological changes in the small intestine tissue of healthy, normotensive rats, in which both 50% and 100% of the traditional CuCO_3_ was replaced by CuNPs. In the histological image of the small intestine of rats receiving the recommended level of Cu in the diet only in the form of CuCO_3_, isolated extensive changes in the base of intestinal villi were found [31]. In light of the above, it can be assumed that the observed damage to the top parts of the intestinal villi may be the result of a change in the rheology of the gastrointestinal content due to the presence of dietary fibre. Dietary fibre, binding water, increases its volume, and thus, the pressure of intestinal contents on the walls of the small intestine. It is therefore highly probable that the movement of the swollen, viscous chyme through successive fragments of the small intestine was accompanied by greater friction, thus leading to damage to the top parts of the intestinal villi.

Numerous reports in the literature indicate that the use of Cu nanoparticles in animal nutrition may be associated with many risks, such as their increased accumulation in internal organs [11,17]. Henson et al. [64], in an in vitro model study, showed that CuO nanoparticles are much more cytotoxic to rat intestinal epithelial cells (IEC-6) than Cu^2+^ ions, and this effect is stronger the higher their dose and the longer the exposure time. The authors observed that increased production of free oxygen radicals resulted in damage to the mitochondrial membrane and reduced viability of small intestine cells exposed to CuO NPs [64]. Lee et al. [17] suggest that the toxicity of copper nanoparticles may be a result of the fact that, in the acidic conditions of gastric juice, they can dissociate to Cu^2+^ ions. However, the authors did not record a similar phenomenon in intestinal juice [17]. Henson et al. also confirmed that there was no release of Cu^2+^ ions from CuO NPs in the intestinal juice [64]. According to the authors, this may indicate that the cytotoxicity of CuO NPs towards intestinal cells is not dependent on their dissociation into the ionic form, but on the specific and natural nature of this form of Cu [64]. The results of our previous studies have also shown that CuNPs are absorbed in the small intestine to a much greater extent than standard forms of this element, such as CuCO_3_ [11], which, in light of the results obtained by Lee et al. [17] and Henson et al. [64], should result in damage to the intestinal barrier and the occurrence of pathological changes in the examined tissue. The results of our study, apart from the possibility of intensifying DNA oxidation by CuNPs, did not confirm this assumption, indicating the highly toxic effect of CuNPs disrupting the integrity of the intestinal barrier, even when used in a dose two times higher than the nutritional recommendations for rats. The introduction of twice as high levels of CuNPs into the diet did not have a significant effect on the increased level of this element in the blood plasma (unpublished data). This effect may therefore be related to the direct effect of dietary fibre introduced into the diet. So far, it has not been possible to clearly determine the effect of different types of dietary fibre on the intestinal absorption of minerals. There are reports indicating both a beneficial and negative effect of dietary fibre [22,23,24,25]. According to Coudray [27], mineral absorption also largely depends on the type and amount of fibre in the diet, as well as mineral homeostasis in the body. Coudray et al. [25] showed an increase in the absorption and retention of Cu in the intestines as a result of inulin consumption. In turn, Krzysik et al. [26] observed a decrease in the absorption of divalent ions as a result of feeding rats a diet containing pectin or cellulose. The decrease in the absorption of trace elements in the small intestine is probably due to the fact that fibre (especially pectin and psyllium) forms a kind of sticky gel in the digestive tract, which is able to bind mainly divalent ions (including Cu^2+^) with free carboxyl groups, which reduces their bioavailability [23,26,65]. Furthermore, pectin may also affect the absorption of minerals by stimulating the bacterial production of short-chain fatty acids (SCFA) and acidifying the intestinal lumen, thus creating unfavourable conditions for the absorption process and intensifying the dissociation of CuNPs to divalent ions [8,26,66]. In addition, the presence of dietary fibre in the diet accelerates the intestinal transit, as a result of which the food content is much shorter in the small intestine, which may significantly reduce the amount of nutrients and minerals absorbed into the body [26,67]. Taken together, it seems that the supplementation of dietary fibre in the form of pectin, inulin and psyllium reduced the absorption of CuNPs, thus protecting the small intestine and modulating its biological response.

## 5. Conclusions

To conclude, replacing CuCO_3_ by CuNPs in the diet of rats positively reduced the level of lactic acid and markers of apoptotic cell death in the small intestine; however, it resulted in the intensification of DNA oxidation. Increasing the level of CuNPs from 6.5 to 13 mg Cu/kg of diet had no negative effect on the physiological intestinal response. Our results indicate that the most visible and beneficial effect on DNA repair mechanisms is related to inulin, while pectin has the greatest ability to inhibit inflammatory processes that induce apoptotic death of cells in the small intestine. The obtained results suggest that dietary fibre supplementation in rats’ diet effectively protects the small intestine against potentially harmful, oxidative effects of CuNPs by intensifying the intestinal barrier, and this may finally translate into a beneficial regulation of their metabolic effect.

## Figures and Tables

**Table 1:** The composition of experimental diets administered to rats for 6 weeks.

	C	CH	CN	CNH	PN	PNH	JN	JNH	SN	SNH
Casein ^1^	14.8	14.8	14.8	14.8	14.8	14.8	14.8	14.8	14.8	14.8
DL-methionine	0.2	0.2	0.2	0.2	0.2	0.2	0.2	0.2	0.2	0.2
Cellulose ^2^	8.0	8.0	8.0	8.0	2.0	2.0	2.0	2.0	2.0	2.0
Pectin					6	6				
Inulin							6	6		
Psyllium									6	6
Choline chloride	0.2	0.2	0.2	0.2	0.2	0.2	0.2	0.2	0.2	0.2
Rapeseed oil	8.0	8.0	8.0	8.0	8.0	8.0	8.0	8.0	8.0	8.0
Cholesterol	0.3	0.3	0.3	0.3	0.3	0.3	0.3	0.3	0.3	0.3
Vitamin mix ^3^	1.0	1.0	1.0	1.0	1.0	1.0	1.0	1.0	1.0	1.0
Mineral mix ^4^	3.5	3.5	3.5	3.5	3.5	3.5	3.5	3.5	3.5	3.5
Maize starch ^5^	64.0	64.0	64.0	64.0	64.0	64.0	64.0	64.0	64.0	64.0
Calculation:										
Cu from, mg/kg										
CuCO_3_	6.5	13	0	0	0	0	0	0	0	0
Cu-NPs	0	0	6.5	13	6.5	13	6.5	13	6.5	13

^1^ Casein preparation: crude protein 89.7%, crude fat 0.3%, ash 2.0% and water 8.0%. ^2^ α-Cellulose (SIGMA, Poznan, Poland), main source of dietary fibre. ^3^ AIN-93G-VM [33], g/kg mix: 3.0 nicotinic acid, 1.6 Ca pantothenate, 0.7 pyridoxine-HCl, 0.6 thiamin-HCl, 0.6 riboflavin, 0.2 folic acid, 0.02 biotin, 2.5 vitamin B-12 (cyanocobalamin, 0.1% in mannitol), 15.0 vitamin E (all-rac-α-tocopheryl acetate, 500 IU g^−1^), 0.8 vitamin A (all-trans-retinyl palmitate, 500,000 IU/g), 0.25 vitamin D-3 (cholecalciferol, 400,000 IU g^−1^), 0.075 vitamin K-1 (phylloquinone), 974.655 powdered sucrose. ^4^ In the experimental treatments with CuNPs, the MX was deprived of CuCO_3_ and, in order to keep the operator safe while preparing the experimental diets, the CuNPs preparation was added as an emulsion, along with dietary rapeseed oil. This procedure was successfully applied in the study authors’ previous experiments. ^5^ Maize starch preparation: crude protein 0.6%, crude fat 0.9%, ash 0.2%, total dietary fibre 0% and water 8.8%.; groups CN and CNH were fed diets with a supplementation of CuNPs (6.5 and 13 mg/kg from Cu-nanoparticles, respectively) with 8% of cellulose dietary fibre source; groups PN and PNH were fed diets with a supplementation of CuNPs (6.5 and 13 mg/kg from Cu-nanoparticles, respectively) with 2% of cellulose and 6% of pectin dietary fibre source; groups JN and JNH were fed diets with a supplementation of CuNPs (6.5 and 13 mg/kg from Cu-nanoparticles, respectively) with 2% of cellulose and 6% of inulin dietary fibre source; groups SN and SNH were fed diets with a supplementation of CuNPs (6.5 and 13 mg/kg from Cu-nanoparticles, respectively) with 2% of cellulose and 6% of psyllium dietary fibre source.

**Table 2:** The primer sequences for analysed genes.

Gene	Primer	Sequence (5′–3′)	Tm (°C)	Product Size (nt)	Gen Bank Access No.
*ACTB*	Forward	CCCGCGAGTACAACCTTCTTG	61,27	71	NM_031144.3
	Reverse	GTCATCCATGGCGAACTGGTG	61,61		
*GAPDH*	Forward	CCGCATCTTCTTGTGCAGTG	59.83	79	NM_017008.4
	Reverse	CGATACGGCCAAATCCGTTC	59.42		
*ZO-1*	Forward	GGAGCGGGGACAAGATGAAG	60.46	123	XM_039105296.1
	Reverse	AGGATGGAGTTACCCACAGC	59.09		
*OCLN*	Forward	GGGGCGCAGCAGGTCT	61.91	181	NM_031329.3
	Reverse	GTGCATCTCTCCGCCATACA	59.90		
*TFF2*	Forward	ACGCCCTCCAACAGAAAGAA	59.53	140	NM_053844.2
	Reverse	CATTGTTCCGACGCTTGGTT	59.41		
*OGG1*	Forward	GACATCGCACCCTAACCTCC	60.18	118	NM_030870.1
	Reverse	CTTTGCTCCCTCCACCGGAA	62.12		

*ACTB*, β-actin; *GAPDH*, glyceraldehyde-3-phosphate dehydrogenase; *ZO-1*, zonula occludens-1; *OCLN*, occludin; *TFF2*, trefoil factor 2; *OGG1*, 8-oxoguanine glycosylase.

**Table 3:** Body weight, feed intake, NMR body composition and internal organs weight in rats fed experimental diets (n = 10 per group) *.

	Initial BW	Final BW	BW Gain	BW Gain	Intake	Heart	Spleen	Kidneys	Body Fat ^^^	Body Lean ^^^	Body Fluids ^^^
	g	g	g	g/day	g/day	g/100 g BW	g/100 g BW	g/100 g BW	%	%	%
Control C	278	396	118	2.91	19.0	0.245	0.183	0.547	13.4	61.6	25.1
Control CH	278	394	116	2.86	18.8	0.243	0.175	0.557	12.7	61.2	24.2
2-way ANOVA:											
CN	278	398	121	2.97	19.1	0.247	0.184	0.542	12.2	62.9	24.9
CNH	278	393	116	2.83	19.1	0.246	0.190 ^&^	0.542	12.9	62.4	24.7
PN	278	395	118	2.86	18.7	0.252	0.181	0.556	12.6	61.9	25.5
PNH	278	398	120	2.92	18.9	0.243	0.181	0.558	13.0	63.0	24.0
JN	278	379	101	2.46 ^#^	17.5 ^#^	0.255	0.202	0.578 ^#^	13.0	62.4	24.6
JNH	278	389	112	2.72	18.3	0.246	0.189 ^&^	0.562	12.8	62.5	24.6
SN	278	385	107	2.58 ^#^	18.4	0.250	0.194	0.573	12.8	63.5	24.1
SNH	278	386	108	2.61	18.2	0.247	0.180	0.564	12.8	62.2	25.0
SEM	1.047	1.725	1.851	0.042	0.110	0.002	0.002	0.004	0.193	0.426	0.503
Cu-NP dose (D)											
L (6.5 mg/kg)	277	389	112	2.72	18.4	0.251	0.190	0.562	12.6	62.7	24.8
H (13 mg/kg)	277	392	114	2.76	18.6	0.246	0.185	0.556	12.9	62.5	24.6
*p* value	0.888	0.559	0.648	0.626	0.346	0.243	0.352	0.544	0.487	0.895	0.882
Fibre type (F)											
C (cellulose)	277	395	118	2.88 ^a^	19.1 ^a^	0.249	0.187	0.542	12.5	62.5	24.8
P (pectin)	278	397	119	2.89 ^a^	18.8 ^ab^	0.247	0.181	0.557	12.8	62.4	24.8
J (inulin)	278	384	107	2.59 ^b^	17.9 ^c^	0.251	0.196	0.570	12.9	62.5	24.6
S (psyllium)	279	386	108	2.59 ^b^	18.3 ^bc^	0.248	0.187	0.569	12.6	62.8	24.5
*p* value	0.998	0.051	0.078	0.028	0.004	0.951	0.322	0.110	0.925	0.991	0.998
Interaction D × F											
*p* value	0.999	0.561	0.575	0.475	0.479	0.778	0.534	0.884	0.917	0.861	0.913

* The dietary treatments used in the experimental feeding period: groups C and CH were fed a control diet with standard and enhanced Cu content in the mineral mixture (6.5 and 13 mg/kg from CuCO_3_, respectively) with 8% of cellulose as a dietary fibre source; groups CN and CNH were fed diets with a supplementation of CuNPs (6.5 and 13 mg/kg from Cu-nanoparticles, respectively) with 8% of cellulose as a dietary fibre source; groups PN and PNH were fed diets with a supplementation of CuNPs (6.5 and 13 mg/kg from Cu-nanoparticles, respectively) with 2% of cellulose and 6% of pectin as a dietary fibre source; groups JN and JNH were fed diets with a supplementation of CuNPs (6.5 and 13 mg/kg from Cu-nanoparticles, respectively) with 2% of cellulose and 6% of inulin as a dietary fibre source; groups SN and SNH were fed diets with a supplementation of CuNPs (6.5 and 13 mg/kg from Cu-nanoparticles, respectively) with 2% of cellulose and 6% of psyllium as a dietary fibre source; L was given treatment (n = 40) with dietary CuNPs 6.5 mg/kg dose; H was given treatment (n = 40) with dietary CuNPs 13 mg/kg dose; C was given treatment (n = 20) with cellulose as dietary fibre; P was given treatment (n = 20) with pectin as dietary fibre; J was given treatment (n = 20) with inulin as dietary fibre; S was given treatment (n = 20) with psyllium as dietary fibre; ^a,b,c^ Mean values within a column with unlike superscript letters are shown to be significantly different (*p* < 0.05); differences between the groups (CN, CNH, PN, PNH, JN, JNH, SN, SNH) are indicated with superscripts only in the case of a statistically significant interaction D × F (*p* < 0.05). Additionally, each experimental group fed CuNPs 6.5 mg/kg (CN, PN, JN, SN) was compared with the control C one with the aid of *t*-test (^#^ indicates a significant difference versus the C group); similarly, each experimental group fed CuNPs 13 mg/kg (CNH, PNH, JNH, SNH) was compared with the control CH with the aid of a *t*-test (^&^ indicates a significant difference versus the CH group); BW, body weight; SEM, pooled standard error of mean (standard deviation for all rats divided by the square root of rat number, n = 100). ^^^ Nuclear magnetic resonance (NMR) analysis.

**Table 4:** Intestinal parameters in rats fed experimental diets (n = 10 per group) *.

	Small Intestine with Contents	Ileal Viscosity	Ileal DM	Ileal pH
	g/100 g BW	mPa·s	%	
Control C	1.37	1.50	19.3	7.14
Control CH	1.38	1.55	19.4	7.15
2-way ANOVA:				
CN	1.33	1.54	19.3	7.22
CNH	1.34	1.51	19.7	7.23
PN	1.49 ^#^	2.72 ^#^	14.3 ^#^	7.01 ^#^
PNH	1.52 ^&^	2.76 ^&^	15.0 ^&^	7.06
JN	1.49 ^#^	1.59	18.1 ^#^	7.15
JNH	1.57 ^&^	1.66	18.0 ^&^	7.09
SN	1.82 ^#^	2.88 ^#^	18.5	7.25 ^#^
SNH	1.78 ^&^	2.95 ^&^	17.6 ^&^	7.23 ^&^
SEM	0.019	0.068	0.217	0.012
Cu-NP dose (D)				
L (6.5 mg/kg)	1.53	2.18	17.5	7.15
H (13 mg/kg)	1.55	2.22	17.6	7.15
*p* value	0.436	0.581	0.937	0.891
Fibre type (F)				
C (cellulose)	1.34 ^c^	1.53 ^b^	19.5 ^a^	7.22 ^a^
P (pectin)	1.50 ^b^	2.74 ^a^	14.6 ^c^	7.03 ^c^
J (inulin)	1.53 ^b^	1.63 ^b^	18.0 ^b^	7.12 ^b^
S (psyllium)	1.80 ^a^	2.91 ^a^	18.0 ^b^	7.24 ^a^
*p* value	<0.001	<0.001	<0.001	<0.001
Interaction D × F				
*p* value	0.323	0.924	0.234	0.463

* The dietary treatments used in the experimental feeding period: groups C and CH were fed a control diet with standard and enhanced Cu content in the mineral mixture (6.5 and 13 mg/kg from CuCO_3_, respectively) with 8% of cellulose as dietary fibre source; groups CN and CNH were fed diets with a supplementation of CuNPs (6.5 and 13 mg/kg from Cu-nanoparticles, respectively) with 8% of cellulose dietary fibre source; groups PN and PNH were fed diets with a supplementation of CuNPs (6.5 and 13 mg/kg from Cu-nanoparticles, respectively) with 2% of cellulose and 6% of pectin as a dietary fibre source; groups JN and JNH were fed diets with a supplementation of CuNPs (6.5 and 13 mg/kg from Cu-nanoparticles, respectively) with 2% of cellulose and 6% of inulin as a dietary fibre source; groups SN and SNH were fed diets with a supplementation of CuNPs (6.5 and 13 mg/kg from Cu-nanoparticles, respectively) with 2% of cellulose and 6% of psyllium as a dietary fibre source; L was given treatment (n = 40) with dietary Cu-NP 6.5 mg/kg dose; H, treatment (n = 40) with dietary CuNPs 13 mg/kg dose; C was given treatment (n = 20) with cellulose as dietary fibre; P was given treatment (n = 20) with pectin as dietary fibre; J was given treatment (n = 20) with inulin as dietary fibre; S was given treatment (n = 20) with psyllium as dietary fibre; ^a,b,c^ Mean values within a column with unlike superscript letters are shown to be significantly different (*p* < 0.05); differences between the groups (CN, CNH, PN, PNH, JN, JNH, SN, SNH) are indicated with superscripts only in the case of a statistically significant interaction D × F (*p* < 0.05). Additionally, each experimental group fed CuNPs 6.5 mg/kg (CN, PN, JN, SN) was compared with the control C with the aid of a *t*-test (^#^ indicates a significant difference versus the C group); similarly, each experimental group fed CuNPs 13 mg/kg (CNH, PNH, JNH, SNH) was compared with the control CH with the aid of a *t*-test (^&^ indicates a significant difference versus the CH group); BW, body weight; SEM, pooled standard error of mean (standard deviation for all rats divided by the square root of rat number, n = 100). Ileal DM, ileal dry matter.

**Table 5:** Blood plasma parameters in rats fed experimental diets (n = 10 per group) *.

	DAO	Lactic Acid
	mIU/mL	ng/mL
Control C	8.63	26.2
Control CH	10.5	22.9
2-way ANOVA:		
CN	8.03	17.3 ^b#^
CNH	9.86	21.6 ^a^
PN	9.20	21.8 ^a#^
PNH	10.2	20.1 ^a&^
JN	9.85 ^#^	21.2 ^a#^
JNH	10.7	20.2 ^a&^
SN	9.67	21.4 ^a#^
SNH	10.9	20.8 ^a^
SEM	0.177	0.314
Cu-NP dose (D)		
L (6.5 mg/kg)	9.19 ^b^	20.5
H (13 mg/kg)	10.4 ^a^	20.7
*p* value	0.001	0.669
Fibre type (F)		
C (cellulose)	8.94 ^b^	19.5
P (pectin)	9.70 ^ab^	21.0
J (inulin)	10.3 ^a^	20.7
S (psyllium)	10.3 ^a^	21.1
*p* value	0.036	0.128
Interaction D × F		
*p* value	0.808	<0.001

* The dietary treatments used in the experimental feeding period: groups C and CH were fed a control diet with standard and enhanced Cu content in the mineral mixture (6.5 and 13 mg/kg from CuCO_3_, respectively) with 8% of cellulose as a dietary fibre source; groups CN and CNH were fed diets with a supplementation of CuNPs (6.5 and 13 mg/kg from Cu-nanoparticles, respectively) with 8% of cellulose as a dietary fibre source; groups PN and PNH were fed diets with a supplementation of CuNPs (6.5 and 13 mg/kg from Cu-nanoparticles, respectively) with 2% of cellulose and 6% of pectin as a dietary fibre source; groups JN and JNH were fed diets with a supplementation of CuNPs (6.5 and 13 mg/kg from Cu-nanoparticles, respectively) with 2% of cellulose and 6% of inulin as a dietary fibre source; groups SN and SNH were fed diets with a supplementation of CuNPs (6.5 and 13 mg/kg from Cu-nanoparticles, respectively) with 2% of cellulose and 6% of psyllium as a dietary fibre source; L was given treatment (n = 40) with dietary CuNPs 6.5 mg/kg dose; H was given treatment (n = 40) with dietary CuNPs 13 mg/kg dose; C was given treatment (n = 20) with cellulose as dietary fibre; P was given treatment (n = 20) with pectin as dietary fibre; J was given treatment (n = 20) with inulin as dietary fibre; S was given treatment (n = 20) with psyllium as dietary fibre; ^a,b^ Mean values within a column with unlike superscript letters are shown to be significantly different (*p* < 0.05); differences between the groups (CN, CNH, PN, PNH, JN, JNH, SN, SNH) are indicated with superscripts only in the case of a statistically significant interaction D × F (*p* < 0.05). Additionally, each experimental group fed CuNPs 6.5 mg/kg (CN, PN, JN, SN) was compared with the control C with the aid of a *t*-test (^#^ indicates a significant difference versus the C group); similarly, each experimental group fed CuNPs 13 mg/kg (CNH, PNH, JNH, SNH) was compared with the control CH with the aid of a *t*-test (^&^ indicates a significant difference versus the CH group); SEM, pooled standard error of mean (standard deviation for all rats divided by the square root of rat number, n = 100). DAO, diamine oxidase.

**Table 6:** Small intestinal biochemical parameters in rats fed experimental diets (n = 10 per group) *.

	APE-1	OGG-1	DAO	8-OHdG	Caspase-3	Caspase-8	Lactic Acid
	ng/g	ng/g	mIU/g	ng/g	ng/g	ng/g	ng/g
Control C	202	85.4	128	21.2	20.9	47.1	5.17
Control CH	195	74.0	121	25.5	16.1	36.2	4.48
2-way ANOVA:							
CN	219 ^a^	89.2	138	28.2^#^	17.0 ^#^	35.1 ^#^	3.56 ^c#^
CNH	205 ^ab^	78.9	131	25.6	16.3	30.4 ^&^	3.81 ^c^
PN	104 ^d#^	77.5	130	22.4	12.3 ^#^	35.7 ^#^	6.24 ^a^
PNH	188 ^bc^	92.1 ^&^	132	23.8	15.6	32.6	4.39 ^c^
JN	142 ^cd^	94.4	133	28.2 ^#^	17.5 ^#^	33.6 ^#^	5.48 ^ab^
JNH	161 ^bc&^	99.7 ^&^	124	27.5	16.0	33.9	5.78 ^a&^
SN	169 ^bc^	96.3	158	25.3	14.5 ^#^	33.5 ^#^	5.32 ^ab^
SNH	196 ^ab^	94.2 ^&^	118	26.9	15.1	39.2	4.13 ^c^
SEM	5.792	1.805	3.779	0.672	0.417	0.942	0.151
Cu-NP dose (D)							
L (6.5 mg/kg)	159	89.4	140	26.0	15.3	34.5	5.15
H (13 mg/kg)	188	91.2	127	25.9	15.8	34.0	4.53
*p* value	0.010	0.634	0.151	0.948	0.584	0.820	0.032
Fibre type (F)							
C (cellulose)	212	84.1 ^b^	135	26.9	16.6 ^a^	32.7	3.69
P (pectin)	146	84.8 ^b^	131	23.1	14.0 ^b^	34.2	5.31
J (inulin)	152	97.1 ^a^	129	27.9	16.8 ^a^	33.8	5.63
S (psyllium)	182	95.3 ^ab^	138	26.1	14.8 ^ab^	36.3	4.72
*p* value	<0.001	0.035	0.898	0.062	0.043	0.642	<0.001
Interaction D × F							
*p* value	0.020	0.151	0.409	0.606	0.186	0.294	0.019

* The dietary treatments used in the experimental feeding period: groups C and CH were fed a control diet with standard and enhanced Cu content in the mineral mixture (6.5 and 13 mg/kg from CuCO_3_, respectively) with 8% of cellulose as a dietary fibre source; groups CN and CNH were fed diets with supplementation of CuNPs (6.5 and 13 mg/kg from Cu-nanoparticles, respectively) with 8% of cellulose as a dietary fibre source; groups PN and PNH were fed diets with supplementation of CuNPs (6.5 and 13 mg/kg from Cu-nanoparticles, respectively) with 2% of cellulose and 6% of pectin as a dietary fibre source; groups JN and JNH were fed diets with supplementation of CuNPs (6.5 and 13 mg/kg from Cu-nanoparticles, respectively) with 2% of cellulose and 6% of inulin as a dietary fibre source; groups SN and SNH were fed diets with supplementation of CuNPs (6.5 and 13 mg/kg from Cu-nanoparticles, respectively) with 2% of cellulose and 6% of psyllium as a dietary fibre source; L was given treatment (n = 40) with dietary CuNPs 6.5 mg/kg dose; H, treatment (n = 40) with dietary CuNPs 13 mg/kg dose; C was given treatment (n = 20) with cellulose as dietary fibre; P was given treatment (n = 20) with pectin as dietary fibre; J was given treatment (n = 20) with inulin as dietary fibre; S was given treatment (n = 20) with psyllium as dietary fibre; ^a,b,c,d^ Mean values within a column with unlike superscript letters are shown to be significantly different (*p* < 0.05); differences between the groups (CN, CNH, PN, PNH, JN, JNH, SN, SNH) are indicated with superscripts only in the case of a statistically significant interaction D × F (*p* < 0.05). Additionally, each experimental group fed CuNPs 6.5 mg/kg (CN, PN, JN, SN) was compared with the control C with the aid of a *t*-test (^#^ indicates a significant difference versus the C group); similarly, each experimental group fed CuNPs 13 mg/kg (CNH, PNH, JNH, SNH) was compared with the control CH with the aid of a *t*-test (^&^ indicates a significant difference versus the CH group); SEM, pooled standard error of mean (standard deviation for all rats divided by the square root of rat number, n = 100). APE-1, apurinic/apyrimidinic endonuclease 1; OGG1, 8-oxoguanine glycosylase; 8-OHdG, 8-hydroxy-2′-deoxyguanosine; DAO, diamine oxidase.

**Table 7:** Level of gene expression in the small intestine of rats fed experimental diets (n = 10 per group) *.

	*OCLN*	*OGG1*	*TFF2*	*ZO-1*
Control C	1.34	1.92	1.15	1.05
Control CH	0.543	0.279	0.306	0.215
2-way ANOVA:				
CN	0.729	0.087 ^b#^	0.326 ^#^	0.407 ^#^
CNH	0.947	0.279 ^ab^	0.306	0.311
PN	0.651	0.719 ^a^	0.458 ^#^	0.468 ^#^
PNH	0.902	0.110 ^b^	0.402 ^&^	0.518 ^&^
JN	0.966	0.346 ^ab#^	0.750	0.337 ^#^
JNH	0.672	0.379 ^ab^	0.361	0.605 ^&^
SN	0.958	0.139 ^b#^	0.305 ^#^	0.761
SNH	1.13 ^&^	0.240 ^b&^	0.511 ^&^	0.646 ^&^
SEM	0.073	0.093	0.052	0.039
Cu-NP dose (D)				
L (6.5 mg/kg)	0.826	0.323	0.460	0.493
H (13 mg/kg)	0.912	0.252	0.395	0.520
*p* value	0.552	0.501	0.501	0.713
Fibre type (F)				
C (cellulose)	0.838	0.183	0.316	0.359 ^b^
P (pectin)	0.776	0.414	0.430	0.493 ^b^
J (inulin)	0.819	0.362	0.556	0.471 ^b^
S (psyllium)	1.04	0.190	0.408	0.704 ^a^
*p* value	0.567	0.289	0.370	0.013
Interaction D × F				
*p* value	0.504	0.037	0.189	0.228

* The dietary treatments used in the experimental feeding period: groups C and CH were fed a control diet with standard and enhanced Cu content in the mineral mixture (6.5 and 13 mg/kg from CuCO_3_, respectively) with 8% of cellulose as dietary fibre source; groups CN and CNH were fed diets with a supplementation of CuNPs (6.5 and 13 mg/kg from Cu-nanoparticles, respectively) with 8% of cellulose as a dietary fibre source; groups PN and PNH were fed diets with a supplementation of CuNPs (6.5 and 13 mg/kg from Cu-nanoparticles, respectively) with 2% of cellulose and 6% of pectin as a dietary fibre source; groups JN and JNH were fed diets with a supplementation of CuNPs (6.5 and 13 mg/kg from Cu-nanoparticles, respectively) with 2% of cellulose and 6% of inulin as a dietary fibre source; groups SN and SNH were fed diets with a supplementation of CuNPs (6.5 and 13 mg/kg from Cu-nanoparticles, respectively) with 2% of cellulose and 6% of psyllium as a dietary fibre source; L was given treatment (n = 40) with dietary CuNPs 6.5 mg/kg dose; H was given treatment (n = 40) with dietary CuNPs 13 mg/kg dose; C was given treatment (n = 20) with cellulose as dietary fibre; P was given treatment (n = 20) with pectin as dietary fibre; J was given treatment (n = 20) with inulin as dietary fibre; S was given treatment (n = 20) with psyllium as dietary fibre; ^a,b^ Mean values within a column with unlike superscript letters are shown to be significantly different (*p* < 0.05); differences between the groups (CN, CNH, PN, PNH, JN, JNH, SN, SNH) are indicated with superscripts only in the case of a statistically significant interaction D × F (*p* < 0.05). Additionally, each experimental group fed CuNPs 6.5 mg/kg (CN, PN, JN, SN) was compared with the control C with the aid of a *t*-test (^#^ indicates a significant difference versus the C group); similarly, each experimental group fed CuNPs 13 mg/kg (CNH, PNH, JNH, SNH) was compared with the control CH with the aid of a *t*-test (^&^ indicates a significant difference versus the CH group); SEM, pooled standard error of mean (standard deviation for all rats divided by the square root of rat number, n = 100). *OCLN*, occluding; *OGG1*, 8-oxoguanine glycosylase; *TFF2*, trefoil factor 2; *ZO-1*, zonula occludens-1.

## Data Availability

All data generated or analysed during this study are included in this published article.
